# Characterization of *lasR*-deficient clinical isolates of *Pseudomonas aeruginosa*

**DOI:** 10.1038/s41598-018-30813-y

**Published:** 2018-09-06

**Authors:** Yao Wang, Leiqiong Gao, Xiancai Rao, Jing Wang, Hua Yu, Junru Jiang, Wei Zhou, Jin Wang, Yonghong Xiao, Mengwen Li, Yan Zhang, Kebin Zhang, Li Shen, Ziyu Hua

**Affiliations:** 1 Department of Neonatology, Children’s Hospital of Chongqing Medical University, Ministry of Education Key Laboratory of Child Development and Disorders, China International Science and Technology Cooperation base of Child development and Critical Disorders, Chongqing Key Laboratory of Child Infection and Immunity, Chongqing, 40014 China; 2 Department of Microbiology, Immunology, and Parasitology, Louisiana State University Health Sciences Center, New Orleans, LA 70112 USA; 3 Department of Microbiology, Army Medical University, Chongqing, 400038 China; 4 Department of Central Laboratory, Xinqiao Hospital, Army Medical University, Chongqing, China; 5 Phase I Clinical Centre, Beijing Shijitan Hospital, Capital Medical University, Beijing, 100038 China; 6 Collaborative Innovation Center for Diagnosis and Treatment of Infectious Diseases, the First Affiliated Hospital, College of Medicine, Zhejiang University, Hangzhou, China 300013

## Abstract

*Pseudomonas aeruginosa* is a prevalent opportunistic pathogen that causes fatal infections in immunocompromised individuals. Quorum sensing (QS) is a cell-to-cell communication process that controls virulence gene expression and biofilm formation in *P*. *aeruginosa*. Here, the QS systems and the relevant virulence traits in clinical *P*. *aeruginosa* isolates were characterized. Eleven out of the ninety-four *P*. *aeruginosa* isolates exhibited a biofilm-deficient phenotype. Two biofilm-deficient isolates, one from blood and the one from pleural effusion, appeared to carry a same mutation in *lasR*. These two isolates differed in the ability to produce QS-regulated virulence factors, but contained the same functionally deficient LasR with the truncated C-terminal domains and belonged to the same multilocus sequence type (ST227). Chromosomal *lasR* complementation in these *lasR* mutants verified that *lasR* inactivation was the sole cause of *las* deficiency. LasR was not absolutely required for *rhl* signal in these *lasR* mutants, suggesting the presence of *lasR*-independent QS systems. We provided evidence that the virulence gene expression are not regulated in the same manner in these isolates. These results support the hypothesis that conventional QS hierarchy can be smashed by naturally occurring *lasR* mutation in clinical *P*. *aeruginosa* isolates and that complex QS hierarchy may play a role in maintaining infection of this opportunistic pathogen.

## Introduction

*Pseudomonas aeruginosa* is a notoriously opportunistic pathogen that causes considerable morbidity and mortality in immunocompromised patients^1,2^. It is particularly dangerous for patients with severe wounds, cystic fibrosis (CF), and cancer. The infection strategy of *P*. *aeruginosa* hinges on the production of numerous cell-associated and secreted molecules, including various proteases and toxins^3–6^. *P*. *aeruginosa* can also form a biofilm that prevents host defenses and increases 10–1000 fold resistance to antimicrobial treatment compared to the same strains in planktonic culture^7,8^.

*P*. *aeruginosa* uses intertwined cell-to-cell signaling or quorum sensing (QS) to sense bacterial cell density, regulate the expression of ~10% of its transcriptome^9,10^, and adapt to biofilm lifestyle^7,8^. Much of our understanding of *P*. *aeruginosa* QS comes from the analysis of laboratory strains, such as PAO1 and PA14^9,11^. Three major QS systems are hierarchically arranged in *P*. *aeruginosa*^12–15^. At the top of the signaling cascade is the acyl-homoserine lactone (AHL)-based *las* system which contains the signal receptor, LasR, its cognate signal molecule or autoinducer 3-oxo-C12-homoserine lactone (OdDHL), and the OdDHL synthase, LasI. The second system is the AHL-based *rhl* system which contains RhlR, its autoinducer C4-homoserine lactone (C4HSL), and the C4HSL synthase, RhlI. The third QS system is the *Pseudomonas* quinolone signal (PQS) involving the binding of the receptor, PqsR, to 2-heptyl-3-hydroxy-4-quinolone^14,16^. The OdDHL-bound-LasR complex serves as a transcriptional regulator to activate the expression of QS genes, including *lasR*, *lasI*, *rhlR*, *rhlI*, and *pqsR*. It also positively regulates the production of virulence factors, such as elastase LasB^17^, exotoxin A^18^, pyocyanin^19^, and extracellular polymeric substrates (EPS)^20^. The latter of which is an important component of biofilm. PQS and the *rhl* system regulate each other^21,22^. Thus, the QS systems control the virulence gene transcription mainly via the master regulator, LasR, and the subordinate regulator, RhlR in *P*. *aeruginosa*.

Bacterial pathogens often require QS to establish and promote infection^23^. Several animal studies have shown the importance of QS systems in the pathogenesis of *P*. *aeruginosa* acute and chronic infections^24–27^. The significance of QS in human *P*. *aeruginosa* infection is less clear, because QS-deficient strains are frequently identified in respiratory acute and chronic infections^28–31^, which raises interesting questions about the necessity of QS in infection. Feltner *et al*.^28^ have reported that 22% of *P*. *aeruginosa* isolates from the lungs of CF patients are mutants and contain polypeptides that differed from LasR in laboratory strains. The presence of *lasR* mutants has been considered to be an adaptation to specific environments, such as the airways of CF patients. An increasing amount of clinical studies has linked *lasR* mutants to worsening disease progression of chronic and acute infections^29,32^. Therefore, better understanding the mechanisms underlying QS-mediated virulence in clinical *P*. *aeruginosa* isolates may aid in full elucidation of the *P*. *aeruginosa* pathogenesis.

The present study aimed at exploring the relationship between QS and the relevant virulence traits in clinical *P*. *aeruginosa* isolates derived from Chinese patients. Specially, two isolates, one from bloodstream and other one from pleural effusion, drew our interest, because they appeared to carry the same mutation in *lasR* and exhibited different virulence properties. Using molecular approaches and biological assays, these *lasR* mutants were characterized in comparison with the well-studied reference *P*. *aeruginosa* strain, PAO1. Our results explained the phenotypic variation observed in these *lasR* mutants and demonstrated that *lasR* deficiency can occur naturally in *P*. *aeruginosa* isolates. It is likely that complex QS hierarchy plays a role in maintaining the infection of this opportunistic pathogen.

## Results

### Phenotypic variation in clinical *P. aeruginosa* isolates

**Figure 1:**
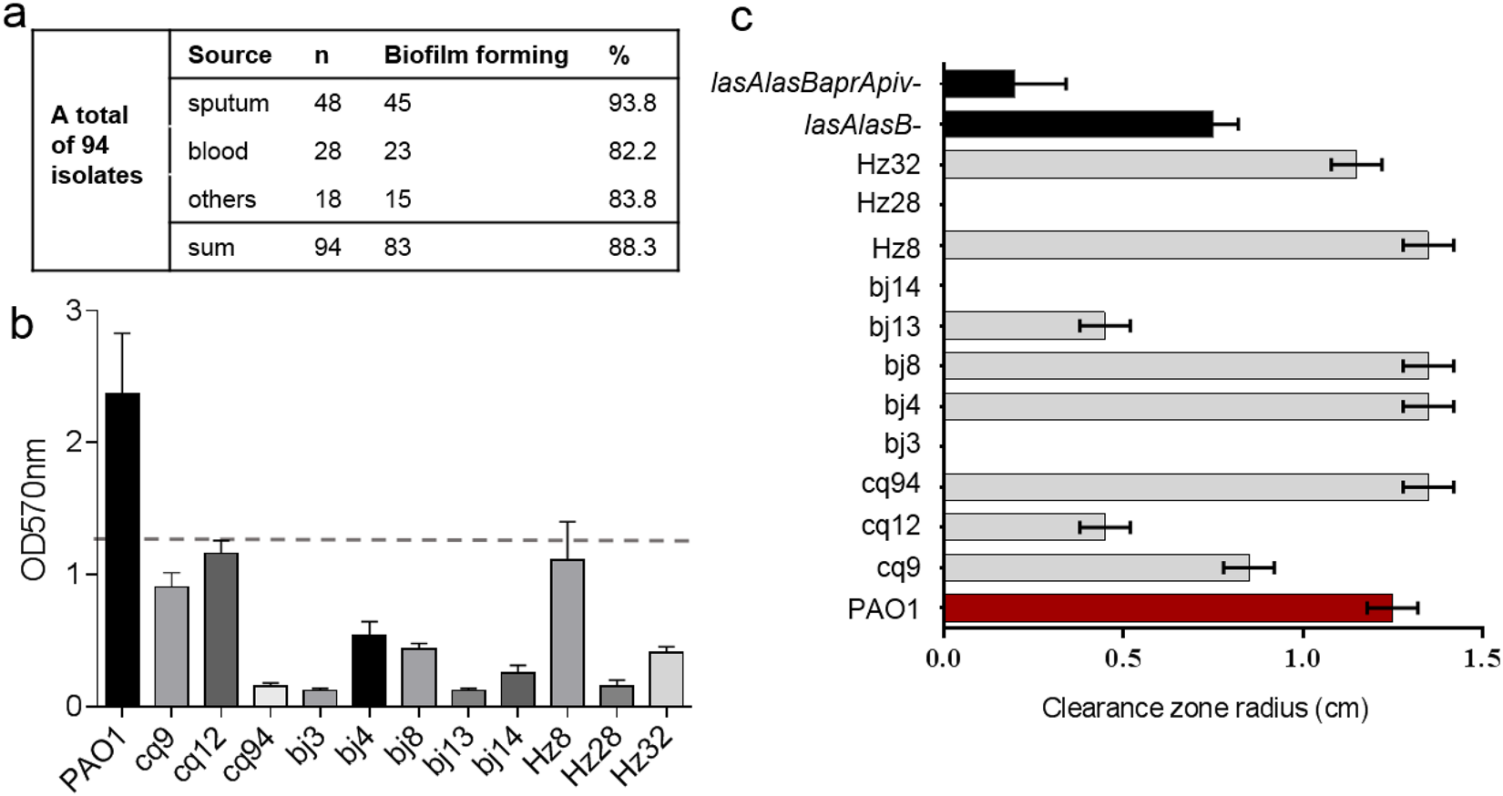
Phenotypic analysis of the clinical *P. aeruginosa* isolates. (**a**) Biofilm forming proficiency of isolates from different sources (see Supplemental Table S1 for details of all clinical isolates). (**b**) Representative results of biofilm forming assays. The values of OD_570_ are presented as means ± standard deviations (SDs) of triplicate assays. The break line indicates OD_570_ value equal to 1/2 of that of PAO1. The isolates had the OD_570_ values ≤ 1/2 of that in PAO1 are defined as the biofilm-deficient. (**c**) Analysis of proteolytic activity using the milk plate assays. Measurement of the radius of the proteolytic zones after incubation at 37  °C for 48 h. Data are the radius values from clinical isolates (gray bar) reported as the mean ± SDs from three individual experiments with triplicates in each experiment. The proteolytic zones produced by strains, PAO1 (red bar) and the isogenic mutants deficient in elastase and/or four proteases (Supplemental Table S2) (black bar) were used as the controls.

With the aim to characterize the QS-regulated virulence properties of clinical *P*. *aeruginosa* isolates, we, first, examined the ability to form biofilm using static biofilm assays^33^. A total of ninety-four *P*. *aeruginosa* isolates derived from varying cases of infections (Supplemental Table S1), including sepsis, ventilation-associated pneumonia, and wound infections, were analyzed. Each isolate was from a single specimen of an individual patient. We found that 83 out of 94 (88.3%) *P*. *aeruginosa* isolates formed a biofilm similar to or thicker than that of PAO1 (Fig. 1a). In contrast, 11 isolates formed defective or no biofilms (Fig. 1b).

We next assessed production of protease, which plays an important role in virulence, in these biofilm-deficient isolates by measuring the proteolytic activity on the milk plates^17^. *P*. *aeruginosa* PAO1 and its isogenic strains deficient in alkaline protease, protease IV, and/or elastase (Supplemental Table S2) were used as the controls. Three different patterns were observed (Fig. 1c): five isolates (cq94, bj4, bj8, Hz8, and Hz32) with strong proteolytic activity, three isolates (cq9, cq12, and bj13) with reduced activity, and three others (bj3, bj14, and Hz28) lacking proteolytic activity. The protease-absence was not fully correlated to the biofilm deficiency. This could be explained by the complex regulatory pathways governing of biofilm formation and/or protease production. Beside QS, other signaling pathways, such as two-component systems, also contribute to biofilm formation. Collectively, survey of the biofilm formation and proteolytic activity revealed the repertoire of phenotypic variation in these clinical isolates, reflecting changes in their biology.

### Genomic variation in QS related genes

**Figure 2:**
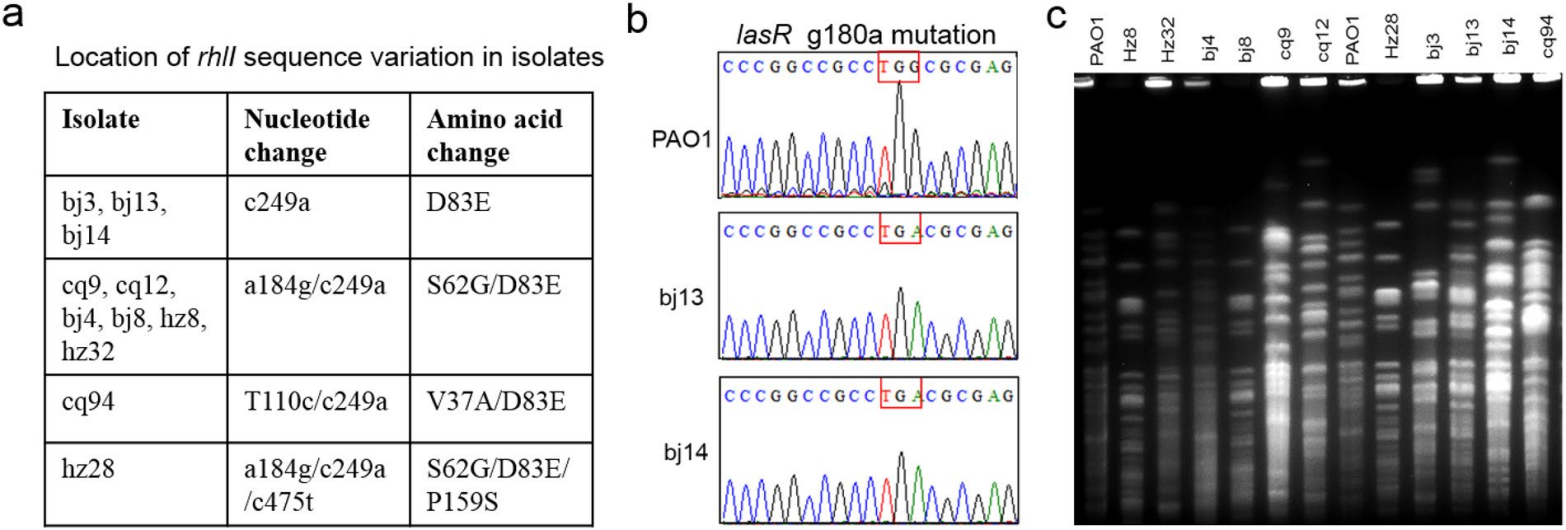
Genotyping of clinical *P. aeruginosa* isolates that displayed a weakened biofilm formation. **(a)** Mutations in *rhlI* gene that resulted in changes in amino acid residues in biofilm-deficient isolates. **(b)** Mutation in *lasR* genes in two isolates, bj13 and bj14. The red box show the changes in the 180^th^ nucleotide in *lasR* leading to LasRW60* mutation. All sequencing results were compared to reference PAO1 genome sequences. **(c)** PFGE pattern of clinical isolates. Genomic DNA extracted from the isolates were digested with SpeI. PAO1 genomic DNA digested with SpeI was used as a control.

We asked whether the biofilm-deficiency phenotype was related to malfunction of the QS. The possession of QS genes, *lasI*, *lasR*, *rhlI*, and *rhlR*, was determined by PCR and DNA sequencing. All biofilm-deficient isolates produced PCR fragments with the predicted molecular sizes (Supplemental Fig. S1). DNA sequencing results showed that the intact *lasI* and *rhlR* genes, same as those in PAO1, were present in all isolates. A few point mutations in *rhlI* were found to alter amino acid residue (s): D83E (in all isolates), S62G (in six isolates), V37A in cq94, and P159S in Hz28 (Fig. 2a). The sequence conflicts with RhlI D83E and S62G in different stock of PAO1 have been reported previously^11,34^. It has been reported the mutation of D83E is unaffected on *rhlI* and its encoded product, C4HSL, in *P*. *aeruginosa* isolates from CF patient^35^. The mutants V37A/D83E or S62G/D83E/P159S might be disruptive to RhlI functionality because at least one of these residues was located in the predicted functional region in RhlI. Moreover, all isolates carried an intact *lasR* gene same as that of PAO1 with the exception of bj13 (from pleural effusion) and bj14 (from bloodstream). Both bj13 and bj14 carried a single mutation at nucleotide 180 (g → a) in *lasR* (Fig. 2b), leading to the change from a tryptophan codon (TGG) to a stop codon (TGA) and premature termination of LasR translation at 60 amino acids. This mutant was designated as LasRW60*.

The analysis of multiple locus sequencing typing (MLST) revealed that bj13 and bj14 had the same allelic profile of 39-5-9-11-27-5-2, corresponding to ST227, a rare ST clone in China. To further distinguish these and other biofilm-deficient isolates, pulsed-field gel electrophoresis (PFGE)^36^ was performed. All biofilm-deficient isolates were successfully typed by PFGE (Fig. 2c). Eight isolates, including bj13 and bj14, displayed distinct PFGE profiles, indicating the different clonality. Three isolates (bj8, Hz8, and Hz28) displayed a similar PFGE pattern. However, these isolates were from different patients and displayed different proteolytic activity (Fig. 1). Thus, each of the isolates was distinct.

Because the *P*. *aeruginosa* isolates were analyzed shortly after isolation without prolonged subculturing, the genotypic and phenotypic variations observed might emerge *in vivo*. With intact *lasI*, *rhlR* and *rhlI*, bj13 and bj14 appeared to have the same LasRW60* mutant and different proteolytic activity. These isolates may have evolved distinct QS circuits to regulate virulence factor production. To test this possibility, bj13 and bj14 were chosen for further study.

### Impaired *las* signal in bj13 and bj14

**Figure 3:**
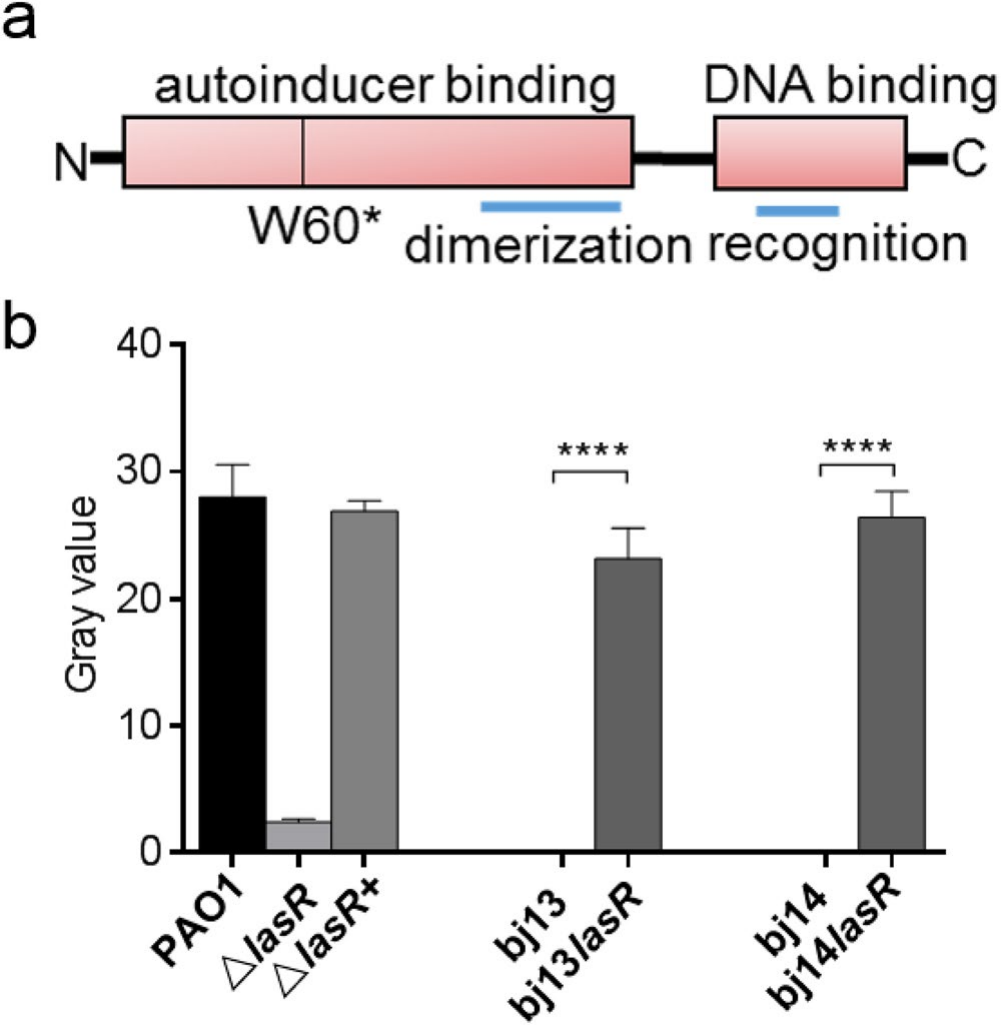
LasR dependence of OdDHL production. **(a**) Schematic diagram shows domain structure of LasR and the location of W60* mutation. (**b**) OdDHL levels assessed by a reporter assay. Extracts of autoinducer from the supernatant of bacterial culture grown for 18 hours were added into the agar plates containing the reporter strains. After incubation at 30 °C for 24 hours, the gray values were measured. The LasR mutants (bj13 and bj14) and the relevant LasR complemented strains (bj13*lasR* & bj14*lasR*) are shown for comparison. PAO1, Δ*lasR*, and the Δ*lasR*+were used as the controls. The gray values are presented as the mean ± SDs of tests performed in triplicates in three different occasions. ****P<0.0001; *P* > 0.05, ns (no significance). *P* values were obtained by one way ANOVA with Bonferroni test.

A functional LasR contained the N-terminal autoinducer binding-dimerization domains and a C-terminal DNA binding domain^37,38^. To address whether the LasRW60* mutant was, indeed, a loss-of-function variant, the levels of OdDHL produced by LasI in bj13 and bj14 were assessed using reporter assays^39^. While PAO1 generated a high level of OdDHL, neither bj13 nor bj14 produced detectable OdDHL, the observation similar to the PAO1-derived *lasR* knockout, Δ*lasR* (Fig. 3b). To confirm that the decreased OdDHL was solely caused by the lack of LasR, we introduced wild-type *lasR* from PAO1 into the chromosome of bj13, bj14, and Δ*lasR* (for a control), respectively. We observed that the OdDHL levels were fully restored in the complemented strains, bj13*lasR*, bj14*lasR*, and Δ*lasR*+(Fig. 3b). These results are consistent with the intact *lasI* that produces OdDHL in the presence of LasR. Thus, loss-of-*lasR*-function was the cause of *las* deficiency in bj13 and bj14.

### Altered *rhl* signal and QS-regulated gene expression in bj13 and bj14

**Figure 4:**
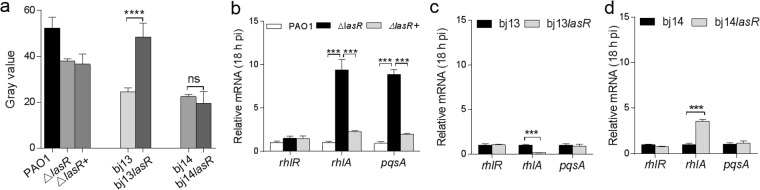
Difference in *rhl*-related gene expression in *P. aeruginosa* isolates. **(a)** C4HSL levels assessed by a reporter assay. The supernatants of *P*. *aeruginosa* culture grown for 18 hours were assayed for C4HSL levels. (**b**–**d**) The relative mRNA levels of *rhlR*,*rhlA* and *pqsA* in *P*. *aeruginosa*. The total RNAs from *P*. *aeruginosa* at the stationary phase were used for real time qRT-PCR analysis. Relative mRNA levels were obtained by normalizing the level of *rhlR*, *rhlA* and *pqsA* to that of the *rplS*. The representative data from the strains with backgrounds of PAO1 (**b**), bj13 (**c**), or bj14 (**d**) were reported as mean ± SD of quadruplicates in an experiment. Experiments were repeated for three times. ****P < 0.0001; *P* > 0.05, ns (no significance). *P* values were obtained by one way ANOVA with Bonferroni test.

We sought to assess the *rhl* signal in these *lasR* mutants by measuring the levels of C4HSL produced by RhlI using reporter assays^39^. It was found that the C4HSL levels in bj13 and bj14 were 39% and 44% of that detected in PAO1 (Fig. 4a), respectively, indicating that RhlI partially functioned in the absence of LasR in these two isolates. Chromosomal complementation with LasR had little effects on C4HSL product in bj14 or PAO1 background. However, the levels of C4HSL increased in strain bj13*lasR* compared to bj13, suggesting a role of LasR in Rhl activation. The failure of restoring for *rhl* signal in strains, bj14*lasR* and Δ*lasR*+, was not due to the poor expression of LasR, as the OdDHL production was restored in the same strains (Fig. 3). These results suggest that the *rhl* signal is not entirely relies on LasR dependent on different strains, consistent with the report by Dekimpe and Déziel^16^.

It is not easy to explain the disparity in C4HSL results observed in strains bj13*lasR* and bj14*lasR* given that both have the intact *rhlI* and *rhlR*. To test whether *rhl*-mediated QS acts differently in bj13 and bj14, real-time qRT-PCR was performed to measure the mRNA levels of *rhlR* and its regulated *rhlA*, in the presence or absence of *lasR*. The *rhlA* is the first gene in *rhlAB* operon encoding rhamnosyl transferases. The *rplS* coding for the 50S ribosomal protein L19 was used as an internal control for quantitation of mRNA levels. We evaluated the growth curve of the isolates compared to PAO1 and found all strains entered into the stationary phase after growing for 10 h and longer in Luria-Bertani (LB) broth (Supplemental Fig. S2). For comparison, mRNA extracted from *P*. *aeruginosa* organisms grown for 6, 12, or 18 h were measured. Both bj13 and bj14 expressed a low level of *rhlR* regardless of the growth phase (Fig. 4 and Supplemental Fig. S3). There was no differences in *rhlR* levels between bj13 and bj13*lasR* or between bj14 and bj14*lasR*, implying little impact of LasR on *rhlR* expression in these strains. Whereas bj13 expressed a higher level of *rlhA* than bj14, the levels of *rhlA* were repressed in bj13*lasR* but increased in bi14*lasR* (Fig. 4 and Supplemental Fig. S3). This change in *rhlA* levels occurred only at the stationary phase, consistent with that QS-based transcriptional responses in *P*. *aeruginosa* increase at high bacterial density.

Curiously, PAO1 assumed as QS proficient expressed little *rhlR* and *rhlA*. Deleting *lasR* caused an increase in *rhlA* and unchanged *rhlR* levels in PAO1 at the stationary phase (Fig. 4b and Supplemental Fig. S3). Neither *rhlA* nor *rhlR* expression was altered in Δ*lasR*+ expressing LasR. Previous studies have shown that, in addition to interlined *las*/*rhl*, PQS can regulate *rhlAB* transcription in PAO1^16^. To confirm this and to explore whether PQS was active in bj13 and bj14, we assessed the expression of *pqsA*, the first gene in the *pqs*ABCDE operon necessary for PQS production. Indeed, the *pqsA* levels were increased in Δ*lasR* lacking LasR coupled to the upregulation of *rhlA* at the stationary phase, suggesting the role of PQS in the activation of *rhlR* in PAO1. However, we failed to detect the similar correlations between *rhlA* and *pqsA* in isolates, bj13 and bj14. Seemingly, PQS did not activate the *rhl* system in bj13 and bj14 background. These results verify that the QS circuits regulating *rhl* in bj13 and bj14 are different from those in PAO1.

### Differences in the production of LasB, exotoxin A, and pyocynain in bj13 and bj14

To address whether QS-controlled virulence gene products are expressed differently in bj13 and bi14, we assessed the bacterial secreted LasB, pyocynain, and exotoxin A in the presence or absence of LasR. These virulence factors affect a variety of *P*. *aeruginosa*-host interactions. While LasB damages host tissue and promotes invasion^17,40^, exotoxin A causes host cell death by blocking protein synthesis^6^. Pyocynain affects the electron transport chain and interferes with host cell growth^19,41^.

**Figure 5:**
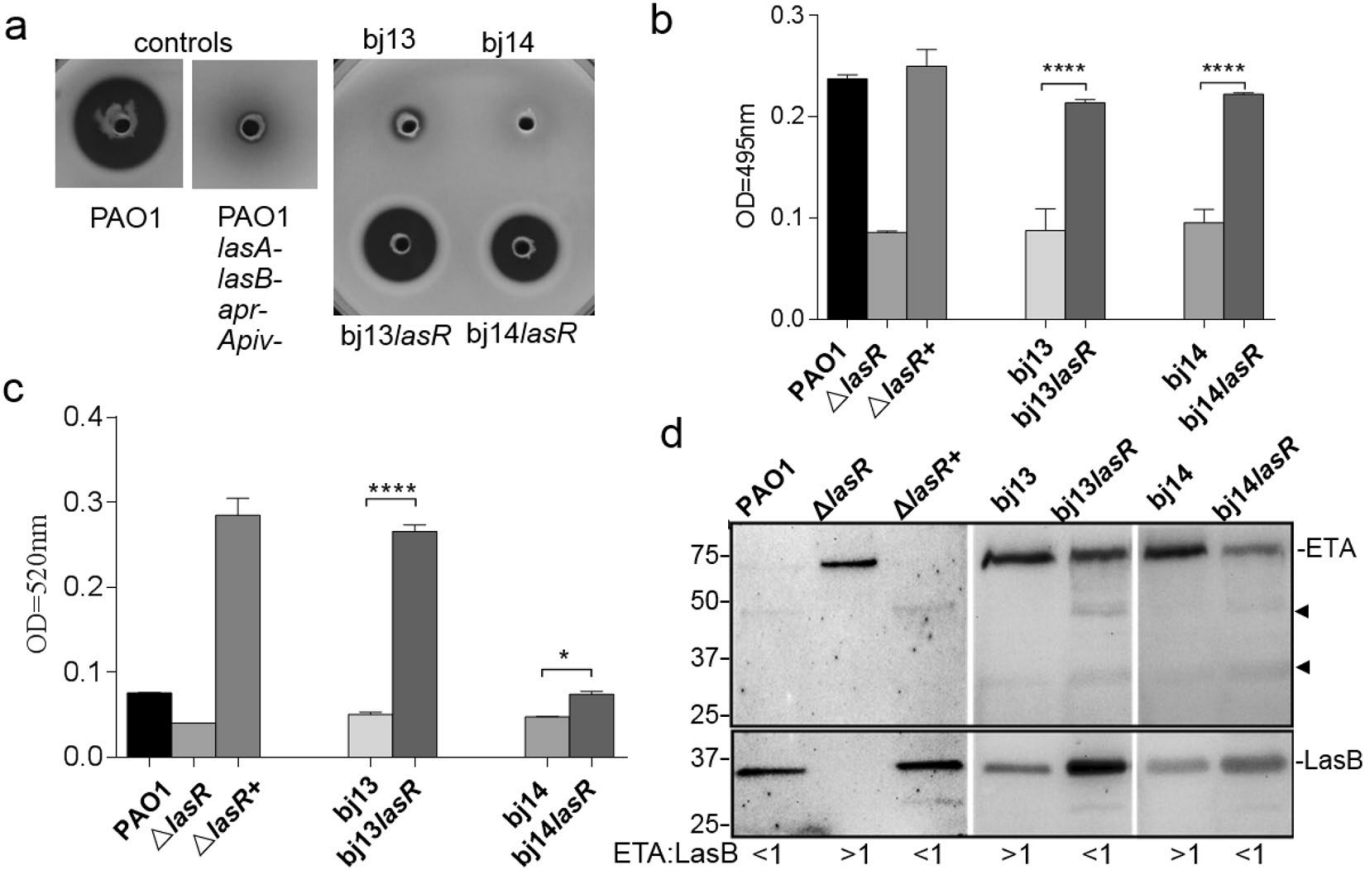
Effects of LasR on production of virulence factors in *P. aeruginosa* isolates. (**a**) Complementation of a *lasR* mutant with the *lasR* restored proteolytic activity. Data represent the lysis zones on milk plate after 24 h of *P*. *aeruginosa* growth at 37 °C. **(b**) Analysis of LasB activity using ECR assays. Cell-free supernatant from bacterial culture grown for 18 hours were used. (**c**) Levels of pyocynain. Extraction of pyocyanin from the supernatant fraction of bacterial cultures grown for 48 hours were measured at 520 nm. Values of each experiment are presented as the mean ± SDs of tests performed in triplicate. Experiments were performed in three different occasions. ****P<0.0001; *P < 0.05. *P* values were obtained by one way ANOVA with Bonferroni test. **(d)** Immunoblotting analysis of secreted exotoxin A (ETA) and LasB. Equal amounts of proteins were loaded and subjected to SDS-PAGE. ETA and LasB were disclosed by anti-ETA antibody and anti-LasB antibody. Arrows show unknown small protein bands crossreacted with anti-ETA antibody. The ratios of ETA levels to LasB levels were shown underneath the blots. The blot of LasB and ExoA in strains PAO1, Δ*lasR*, and Δ*lasR*+were in the same gel. Strains bj13, bj13*lasR*, bj14 and bj14*lasR* were in the same gel and the full-length blots were shown in Supplemental Figure S5.

Whereas the ability to produce LasB and pyocyanin was impaired in all *lasR* mutants, the activity was restored to a certain degree in *lasR*-complemented strains (Fig. 5a–c). The weak LasB elastinolytic activity in the culture supernatants from bj13 and bj14 (Fig. 5a) was not due to *lasB* mutation in these isolates. Despite two nucleotide mutations in *lasB* resulting in S241G and S435L in bj13 and bj14, none of these changes were at the active site for LasB function as reported previously^42^ (Supplemental Fig. S4). LasB with natural variant S241G have been found in *P*. *aeruginosa* strains, PA103 and N-10. Additionally, we noted that the *lasR*-induced increase in pyocyanin levels in bj13*lasR* was much higher than that in bj14*lasR* (fold changes ~5 *vs* 1.8 times) (Fig. 5c), indicating that regulation of pyocyanin production was different in these isolates. Moreover, a full length exotoxin A (69 kDa) band was readily detected form the cultural supernatant of bj13 and bj14 using immunoblotting analysis (Fig. 5d). Interestingly, in the presence of LasR, this exotoxin A band was less intense coupled to appearance of several faint, smaller antibody reactive bands. At the same time, more intense mature LasB band was detected in the same sample. Exotoxin A was also easily detected from strain, Δ*lasR* lacking *lasR*, but little was detected in PAO1 and Δ*lasR*+. It was unclear if these faint bands were the degraded product of the exotoxin A in the presence of the high levels of proteases, such as LasB. Adding bacterial protease inhibitors did not prevent the appearance of these faint bands. Together with the transcriptional studies described in Fig. 4, these data support that virulence factor production is regulated in different manners in isolates, bj14 and bj13.

## Discussion

To study QS and its regulated virulence factor expression, we started with survey of biofilm formation and protease production with clinical *P*. *aeruginosa* isolates. The screened biofilm-deficient isolates were then analyzed for the possession of QS-related genes and the genotypes. Finally, two unusual isolates, bj13 from pleural effusion and bj14 from bloodstream, were selected because of carrying the same LasRW60* mutant and intact *lasI*, *rhlI*, and *rhlR* genes. Despite the same *lasR* allele and the identical MLST, bj13 and bj14 exhibited clearly different PFGE patterns and virulence traits. We reasoned that bj13 was dissimilar to bj14. Their virulence properties were further characterized.

Of importance, we found that the virulence gene expressions at transcriptional level is regulated in the different manner in these isolates. Consistent with these observations, there were notable differences in the virulence products (i.e., protease, LasB, pyocyanin, and exotoxin A). Although LasR is known as a master positive virulence regulator in *P*. *aeruginosa*^12–15^, crossingregulation between the *las* and *rhl* systems were observed in these isolates. For example, the production of low but detectable C4HSL in the absence of LasR; as such, detection of C4HSL alone is not a reliable proxy for RhlR activity. Additionally, the lack of correlation between *pqsA* and *rhlA* levels suggest that PQS is unlikely the major regulator of the *rhl* system in these isolates. These observations reinforce that the complex QS circuits important for virulence factor expression in these isolates are different from that in PAO1. Previous studies of clinical isolate have demonstrated the *las* system is not necessarily crucial in some occasions^28,29,32,35,43,44^. The LasR-deficient *P*. *aeruginosa* isolates are prevalent in the lung of CF patients^28^ and the individuals with acute ventilation-associated pneumonia^45^. It is known that *P*. *aeruginosa* can genetically adjust the QS hierarchy and the relevant lifestyle when they colonize and infect at the epithelial cells^28,46^ or shift from acute to chronic mode of infections^44^. Loss of *lasR* control might give *P*. *aeruginosa* a selective advantage in pathogenic interactions with the hosts and minimize fitness cost at a bacterial population level. Deficiency in *lasR* may also have been offset by other highly interactive QS systems and/or global regulators, such as Vfr, VqsR, MvaT, GacA, RpoN, RpoS, and RsmA^12^. The presence of *rhl* signal without *las* signal in clinical isolates suggest that the use of *las-*independent QS activity may play a role in maintaining *P*. *aeruginosa* infection.

Many factors could directly contribute to multicellular adaptation and diversities in bacteria^47^. For example, patient immune response, antibiotic therapy, bacterial interspecies or interkingdom competition in microbiome, and the duration of bacterial infections. Selections of *lasR*-inactivation associated with the chronic CF airway infections^28,35^ and the more acute ventilator-associated pneumonia^30^ have been well studied. It is unknown what selection force are for losing *lasR* in bj13 and bj14. Since these isolates are originated from bloodstream and inflammatory pleural fluid, it is possible that exposure to host immunity and environmental factor (such as antibiotics) could be involved. Clinical studies have shown that *P*. *aeruginosa* may enhance the expression of some virulence genes and reduces the expression of others as part of its survival strategy. Hammond *et al*.^32^ reported that *lasR* inactivation was associated with the increased production of CupA fimbriae in the isolates from the patients with acute keratitis. We observed the decreased production of the total protease and LasB coupled to the high steady-state-levels of exotoxin A in *lasR* mutants. Thus, exploring whether the presence of steady-state-levels of exotoxin A in the *lasR* mutants are relevant in *P*. *aeruginosa* pathogenesis *in vivo* will be an interesting future study. Kruczek *et al*.^9^ revealed that *P*. *aeruginosa* strain, PA14, cultured with blood from severe burned patients displayed an altered transcriptome. Transcripts from QS genes and QS-regulated virulence factors were significantly downregulated. Conversely, genes encoding the type III secretion (T3S) system were increased, consistent with the previous reports indicating that the T3S effector is more important in the pathogenesis of the acute infections^48^. Whether bj13 and bj14 carrying *lasR* mutants utilize T3S to compensate for the reduced virulence factors during infection *in vivo* remains to be determined.

Our work is limited in scope but the findings suggest that additional investigation of the important QS networks in clinical isolates may produce fruitful advancements towards helping understanding *P*. *aeruginosa* pathogenesis. Central to *P*. *aeruginosa’s* success as an opportunistic pathogen is the genetic plasticity and the great metabolic capabilities provided by its large genome. To thoroughly identify the pathogenic determinants in bj13 and bj14, characterization of their genetic natures by whole genome sequencing is warranted. Our data did not reveal whether specific *lasR* mutants were associated with a particular patient population or type of diseases. Large sampling, prospective, longitudinal studies of the clinical isolates are needed to gain deeper insights into the evolution dynamics of *P*. *aeruginosa*. Nevertheless, the emergency of the *lasR* mutant in clinical isolates suggests that the *las* system, while important, is not absolutely essential for the establishment of *P*. *aeruginosa* infection *in vivo*. It also calls into question the previously accepted hierarchical understanding of the QS systems which apparently are much multifaceted in the clinical contexts.

## Methods

### Ethics Statement

This study was approved by the Institutional Review Board of Children’s Hospital, Chongqing Medical University (Approval No. 2017-61-1). Given that the data were collected and interpreted anonymously, the need for written informed consent from patients or their legal guardians was waived by the ethics committee.

## Bacteria organisms

A total of 94 *P*. *aeruginosa* strains were isolated from patients admitted for treatment from 2011 to 2013 to three university teaching hospitals located in different cities. These isolates represented one-time isolates from individual patients receiving antimicrobial therapy. The source of the isolates are listed in Supplemental Table S1. They included 48 from sputa, 28 from blood, 11 from pus or skin wounds, 2 from peritoneal fluid, 2 from gall gladder effusion, 1 from pleural effusion, 1 from synovial fluid, and 1 from urine. Broth cultures of isolates were aliquoted to avoid repeated sub-culturing and then were stored at −80 °C. The two main clinical *P*. *aeruginosa* isolates, bj13 and bj14, and other bacteria strains used for detailed molecular analysis were list in Supplemental Table S2. *P*. *aeruginosa* strain, PAO1^40^ and the isogenic strains deficient in alkaline protease, protease IV, and/or elastase were used for controls. Competent *Escherichia coli* DH5α cells were used as the host cells for molecular cloning. Bacteria were cultured in LB broth or LB agar plate at 37 °C supplemented with antimicrobials when appropriate. For selection of *P*. *aeruginosa* transformants, chloramphenicol 600 μg/ml, spectinomycin 600 μg/ml, or tetracycline 600 μg/ml were used. For other experiments, we used carbenicillin 100 μg/ml, tetracycline 30 μg/ml, chloramphenicol 25 μg/ml, and spectinomycin 50 μg/ml.

### Plasmid, mutant construction, and complementation of mutants

The plasmids and primers used are listed in Supplemental Tables 2 and 3, respectively. To create plasmid pUCPRedCm, the Ahd I-Nar I fragment of pARC^49^ containing the promoter region and the coding region of chloramphenicol acetyltransferase was cloned into pUCP-Red at Ahd I-Nar I sites. Plasmids were transformed into *P*. *aeruginosa* by electroporation^39^. The bacteriophage lambda (λ) Red recombinase system^50^ was used for *lasR* mutant creation and complementation. To generate a *lasR* knockout in PAO1 background, the DNA fragment containing tetracycline resistant gene (*tet*) flanked by the 50 bp of homology regions to the target sequence was obtained by polymerase chain reaction (PCR) with primers DLRF/DLRR using plasmid pBR322 as the template. The purified DNA fragment was electroporated into PAO1, bj13 and bj14 carrying pUCPRedCm, resulting in strain Δ*lasR*, bj13Δ*lasR* and bj14Δ*lasR*. For chromosomal *lasR* complementation studies, a DNA fragment containing the cassette of *lasR-aadA* (conferring resistance to streptomycin and spectinomycin) flanked by 50 bp of homology regions to the target sequence was generated by overlap extension PCR with multiple steps. The *lasR*-containing DNA fragment was obtained from PAO1 genomic DNA with primers CLRF/CLRR. The *aadA* containing fragment was amplified from pCDFScc4^51^ with primers CSPF/CSPR. Following the annealing of these two DNA fragments, the full-length *lasR*-*aadA* cassette was amplified with primers CLRF/CSPR, purified, and electroporated into strain Δ*lasR*, bj13Δ*lasR* and bj14Δ*lasR*; each of which carried the pUCPRedCm. The resultant complemented strains were designated as Δ*lasR*+, bj13*lasR* or bj14*lasR*. All gene regions of interest in the constructs were confirmed by DNA sequencing with primers PGDF and PGFR.

### Bacterial genomic analysis

The QS-related genes, *lasB*, *lasR*, *lasI*, *rhlR*, and *rhlI*, were amplified by PCR with bacterial genomic DNA as the templates using appropriate primers (Supplemental Table S3). The purified PCR products were sequenced. Multilocus sequence typing (MLST) was conducted as proposed previously^52^. The fragments of the seven different central housekeeping genes, *acsA*, *aroE*, *guaA*, *mutL*, *nuoD*, *ppsA*, and *trpE*, were amplified by PCR using appropriate primers (Supplemental Table S3). The resulting PCR products were purified and subjected to DNA sequencing. The database at http://pubmlst.org was used to assign numbers to particular alleles and to identify sequence types. Pulsed-field gel electrophoresis (PFGE) was performed using *P*. *aeruginosa* genomic DNA digested with restriction enzyme, *Spe* I, as described previously^36^. All sequence comparison and primers design were based on the PAO1 genome sequence (www.pseudomonas.com).

### Biofilm formation assays

A static microplate biofilm assay^33^ was performed in a polystyrene 96-well plate (Corning). Overnight cultures of *P*. *aeruginosa* were standardized to be diluted to OD_600_ = 0.5 in fresh LB to ensure that equal numbers of bacteria were present in each well. Bacteria were grown in triplicate in a 96-well plate at 37 °C for 48 hours without agitation. *P*. *aeruginosa* strain PAO1 was inoculated on each plate as a positive control and LB served as a blank. Plates were washed three times in distilled water to remove unattached bacteria. Wells were stained with 0.05% crystal violet (Sigma) for 10 min, then washed twice with water. Adherent stain was dissolved in 30% acetic acid for 15 min and then measured at 570 nm. The values of biofilm staining was determined by (OD_570_ sample−OD_570_ LB blank).

### Proteolytic activity, LasB, and pyocyanin assays

Total proteolytic activity was determined using milk plate assays^17^. The LasB activity was measured by an Elastin Congo red (ECR) assay^41^. Bacteria cells were grown in LB broth at 37 °C for 18 h and removed by centrifugation. The supernatant fractions passed through a 0. 22 mm filter were subjected to ECR assay. The reaction liquids were centrifuged and then measured at 495 nm. Pyocyanin was extracted from the supernatant fraction of bacterial cultures grown for 48 hours and measured at 520 nm^53^.

### Autoinducer measurement

The bioluminescence-based reporter assays were performed as described previously^39^. *E*. *coli* reporter strains, JM109/pSB1075 and JM109/pSB536^54^ (Supplemental Table S2) were used to measure the levels of OdDHL and C4HSL, respectively. The supernatant of *P*. *aeruginosa* culture grown for 18 hours was obtained for autoinducer extraction with ethyl acetate (acidified with 0.5% formic acid). The autoinducer containing extracts were dissolved in 50% methanol. Then, the 1:10 dilution of extracts in methanol was pipetted into LB agar plate containing reporter strains. After incubation at 30 °C for 24 h, the gray value formed were measured.

### Real-time reverse transcription quantitative PCR (RT-qPCR)

*P*. *aeruginosa* grown in LB broth were collected after 6, 12, and 18 hours of growth. Total RNA was isolated using the RNA MiniPrep Kit (Zymo) according to the manufacturer’s instructions. DNA-free RNA (1 μg) was reverse transcribed into cDNA with 20 ng random hexamer primers using the high capacity cDNA reverse transcription Kit (Applied Biosystems). Amplification and quantification of cDNA were performed using the VeriQuest Fast SYBR Green qPCR Master Mix (USB). Primer pairs (Supplemental Table S3) with the amplicon sizes of 150 to 200 bp were designed for genes, *rhlR*, *rhlA*, *pqsA*, and *rplS* (as an internal control) using the Primer3 software (http://frodo.wi.mit.edu/cgi-bin/primer3/primer3.cgi/primer3_www.cgi). The qPCR cycle conditions were as follows: 50 °C for 2 min, 95 °C for 2 min, 95 °C for 30 s, 59 °C for 1 min. The latter two steps were repeated for 30 cycles, and fluorescence was detected at the end of each cycle. The 2^−ΔΔ*CT*^ threshold cycle (*C*_*T*_) method was used to calculate the relative transcript levels of genes studied compared to *rplS* transcripts. Note, qPCR reactions were setup quadruplicates and the experiments were performed three times.

### Western blot analysis

The supernatants of *P*. *aeruginosa* culture grown for 18 h (stationary phase) were obtained by centrifugation. The total proteins in supernatants were determined using BCA assays (ThermoFisher). Protein samples in SDS loading buffer containing β-mercaptoethanol were boiled at 98 °C for 5 minutes. Equal amounts of protein were loaded and separated by 10% SDS-PAGE, followed by transferring to a PVDF membrane. A rabbit polyclonal antibody specific to *Pseudomonas* exotoxin A (Sigma, catalog # P2318) or LasB^55^ were used to probe exotoxin A or LasB, respectively. The probed membranes were incubated with anti-rabbit horseradish peroxidase-conjugated IgG at room temperature for 1 hour and 30 min and developed using SuperSignal West Pico chemiluminescent substrate (Pierce).

### Statistical analysis

Statistical analysis of data was conducted using GraphPad Prism 5.0 (La Jolla, CA, USA). A *P* value < 0.05 was considered significant.

## Electronic supplementary material

Supplemental Information
