# Supplementary material for: Characterization of lasR-deficient clinical isolates of Pseudomonas aeruginosa
Source: Sci Rep. 2018 Sep 6;8:13344. doi: 10.1038/s41598-018-30813-y (PMC6127196; doi:10.1038/s41598-018-30813-y)
Supplement: Supplementary file 1 — Supplemental Information [file 41598_2018_30813_MOESM1_ESM.doc]

**Supplemental Information**

**Characterization of *lasR*-deficient clinical isolates of *Pseudomonas aeruginosa***

Yao Wang, Leiqiong Gao, Xiancai Rao, Jing Wang, Hua Yu, Junru Jiang, Wei Zhou, Jin Wang, Yonghong Xiao, Mengwen Li, Yan Zhang, Kebin Zhang, Li Shen, and Ziyu Hua

**Supplemental Tables**

**Table S1.** *P. aeruginosa* isolates used in this study

**Table S2.** Bacteria strains and plasmids used in this study.

**Table S3.**Primers used in this study

**Supplemental figures**

**Figure S1** Agarose gel electrophoresis of PCR products of QS related genes.

**Figure S2** The growth curve of *P. aeruginosa*.

**Figure S3** The growth-phase differential expression pattern of *rhlR*, *rhlA* and *pqsA* genes in *P. aeruginosa* with different background*.*

**Figure S4** The alignment of LasB amino acid sequences in bj13 and bj14

**Figure S5.** The full-length blot of LasB and ExoA in bj13, bj13*lasR*, bj14 and bj14*lasR*.

**References**

**Table S1 *Pseudomonas aeruginosa* isolates used in this study**

| Strains | Source | Strains | Source | strains | source |
| --- | --- | --- | --- | --- | --- |
| bj1  bj2  bj3  bj4  bj5  bj6  bj7  bj8  bj9  bj10  bj11  bj12  bj13  bj14  cq1  cq2  cq3  cq4  cq5  cq6  cq7  cq8  cq9  cq10  cq12  cq13  cq14  cq15  cq16  cq17  cq18 | blood  blood  synovial fluid  blood  blood  pus  blood  blood  blood  blood  blood  blood  pleural effusion  blood  sputum  sputum  sputum  sputum  sputum  sputum  urine  sputum  sputum  sputum  sputum  sputum  sputum  sputum  pus  sputum  sputum | cq19  cq20  cq21  cq22  cq23  cq24  cq25  cq26  cq27  cq28  cq29  cq30  cq31  cq32  cq33  cq34  cq35  cq36  cq37  cq38  cq39  cq40  cq41  cq42  cq43  cq44  cq45  cq46  cq91  cq94  cq95 | sputum  sputum  sputum  sputum  pus  sputum  skin wounds  sputum  sputum  sputum  sputum  sputum  sputum  pus  sputum  sputum  sputum  sputum  sputum  sputum  sputum  sputum  sputum  pus  sputum  sputum  sputum  sputum  skin wounds  skin wounds  skin wounds | Hz1  Hz2  Hz3  Hz4  Hz5  Hz6  Hz7  Hz8  Hz9  Hz10  Hz11  Hz12  Hz13  Hz14  Hz15  Hz16  Hz17  Hz18  Hz19  Hz20  Hz21  Hz22  Hz23  Hz24  Hz25  Hz26  Hz27  Hz28  Hz29  Hz30  Hz31  Hz32 | blood  blood  blood  blood  blood  blood  blood  blood  blood  blood  skin wounds  skin wounds  blood  blood  blood  blood  blood  blood  gall gladder effusion  sputum  sputum  gall gladder effusion  sputum  peritoneal fluid  sputum  sputum  sputum  sputum  sputum  peritoneal fluid  sputum  blood |

**Table S2 Bacterial strains and plasmids used in this study**

| **Strain** | **Description** | **Source** |
| --- | --- | --- |
| ***P. aeruginosa*** |  |  |
| PAO1 | wild type laboratory adapted strain | [^1^](#_ENREF_1) |
| Δ*lasR* | *lasR* deletion derived from PAO1, Cam^r^, Tet^r^ | this study |
| Δ*lasR*+ | Δ*lasR* carrying *lasR*-*aadA* cassette at chromosome, Cam^r^ , Spe^r^ | this study |
| PAO1*lasAlasB-* | deficiency in elastases, LasA and LasB | [^1^](#_ENREF_1) |
| PAO1*lasAlasBaprApiv-* | deficiency in LasA, LasB, alkaline protease, and protease IV | [^1^](#_ENREF_1) |
| bj13 | clinical isolate from pleural effusion | this study |
| bj14 | clinical isolate from bloodstream | this study |
| bj13*lasR* | bj13 carrying *lasR*-*aadA* cassette at chromosome | this study |
| bj14*lasR* | bj14 carrying *lasR*-*aadA* cassette at chromosome | this study |
| ***Escherichia coli*** |  |  |
| JM109/pSB1075 | *lasR + lasI::luxCDABE,* Tet^r^ |  |
| JM109/pSB536 | *rhlR + rhlI::luxCDABE,* Amp^r^ |  |
| DH5α | F^-^ Φ80*lac*ZΔM15 Δ(*lac*ZYA-*arg*F) U169 *rec*A1 *end*A1 *hsd*R17(r_k_^-^, m_k_^+^) *pho*A *sup*E44 *thi*-1 *gyr*A96 *rel*A1 λ^-^ | Invitrogen |
| **Plasmid** |  |  |
| pUCP-Red | *E. coli*-*P. aeruginosa* shuttle vector expressing λ Red controlled by an arabinose-inducible promoter, Amp^r^ | [^4^](#_ENREF_4) |
| pACR | A plasmid containing chloramphenicol resistance gene | [^5^](#_ENREF_5) |
| pUCPRedCm | A shuttle plasmid derived from pUCP-Red, Cam^r^ | this study |
| pBR322 | A plasmid containing tetracycline resistance gene | [^6^](#_ENREF_6) |
| pCDFScc4 | A plasmid containing spectinomycin resistance gene | Novogen |

**Table S3** Primers used in this study

| **Primer** | | **Primer sequence (5’- 3’)** | **Description** |
| --- | --- | --- | --- |
| lasI-F | ATATAGGGAAGGGCAGGTTC | | PCR &sequencing *lasI* |
| lasI-R | GTTCATCGAAGCGGTCTATC | |
| lasR-F | CGTACTAGGTGCATCAAACG | | PCR & sequencing *lasR* |
| lasR-R | GACCTGAGAGGCAAGATCAG | |  |
| rhlI-F | CTCCTTTAGTCTTCCCCCTCAT | | PCR & sequencing *rhlI* |
| rhlI-R | GAAACGGCTGACGACCTC | |  |
| rhlR-F | TTCATGGAATTGTCACAACC | | PCR & sequencing *rhlR* |
| rhlR-R | AGCTTCTCGATGAAGACCTG | |  |
| PRDF | AGTGACTCCCCGTCTTACGCCCCGCCCTG | | constructing pUCPRedCam |
| PRDR | CTAGGCGCCTGATCGGCACGTAAGAGG | |
| DLRF | TATCGGGTGCCGAATCCATATTTGGCTGATTGGTTAATAGTTTAAGAAGAACGTAGCGCTTTGACAGCTTATCAT | | constructing strain, *ΔlasR* |
| DLRR | TGCATTTCTATATAGAAGGGCAAATTACCGATCGCCAGCTCGCCGACCTGAGAGGCAAGATCAGGTCGAGGTG | |
| CLRF | CGAATCCATATTTGGC | | constructing strains, Δ*lasR+*, bj13*lasR* and bj14*lasR* |
| CLRR | TCAGAGAGTAATAAGACCCA | |
| CSPF | CCCGCCGCGTAGCGGCCATTATGGCCGTTAATTTGGGTCTTATTACTCTCTGATTTGTTTATTTTTCTA | |
| CSPR | TATAGAAGGGCAAATTACCGATCGCCAGCTCGCCGACCTGAGAGGCAAGATTATTTGCCGACTAC | |
| PGDF | CGTTTGCTTACCCTCTAGG | | sequencing *lasR* |
| PGDR | AGAACACAGCCCCAAAAC | |  |
| rhlRrtF | GTTGCATGATCGAGTTGCTGAC | | RT-qPCR |
| rhlRrtR | TGGATGTTCTTGTGGTGGAAGT | |
| rplSrtF | GTGAAGGAAGGAGACCGTCA | | RT-qPCR |
| rplSrtR | GCGGAGGTAGTACAGCTTGG | |
| rhlArtF | CTGAAAGCCAGCAACCATC | | RT-qPCR |
| rhlArtR | GGCGGTGGTGTATTCGTC | |
| pqsArtF | CAATACACCTCGGGTTCCAC | | RT-qPCR |
| pqsArtR | TGCCATAGCCGAAGAACATC | |  |
| acsA-F | ACCTGGTGTACGCCTCGCTGAC | | MLST |
| acsA-R | GACATAGATGCCCTGCCCCTTGAT | |
| aroE-F | TGGGGCTATGACTGGAAACC | | MLST |
| aroE-R | TAACCCGGTTTTGTGATTCCTACA | |
| guaA-F | CGGCCTCGACGTGTGGATGA | | MLST |
| guaA-R | GAACGCCTGGCTGGTCTTGTGGTA | |
| mutL-F | CGCGACCTGTTCTTCAACAC | | MLST |
| mutL-R | CAGGGTGCCATAGAGGAAGTC | |
| nuoD-F | ACGGCGAGAACGAGGACTAC | | MLST |
| nuoD-R | TCTCGCCCATCTTGACCA | |
| ppsA-F | GGTCGCTCGGTCAAGGTAGTGG | | MLST |
| ppsA-r | GGGTTCTCTTCTTCCGGCTCGTAG | |  |
| trpE-F | GCGGCCCAGGGTCGTGAG | | MLST |
| trpE-R | CCCGGCGCTTGTTGATGGTT | |

**
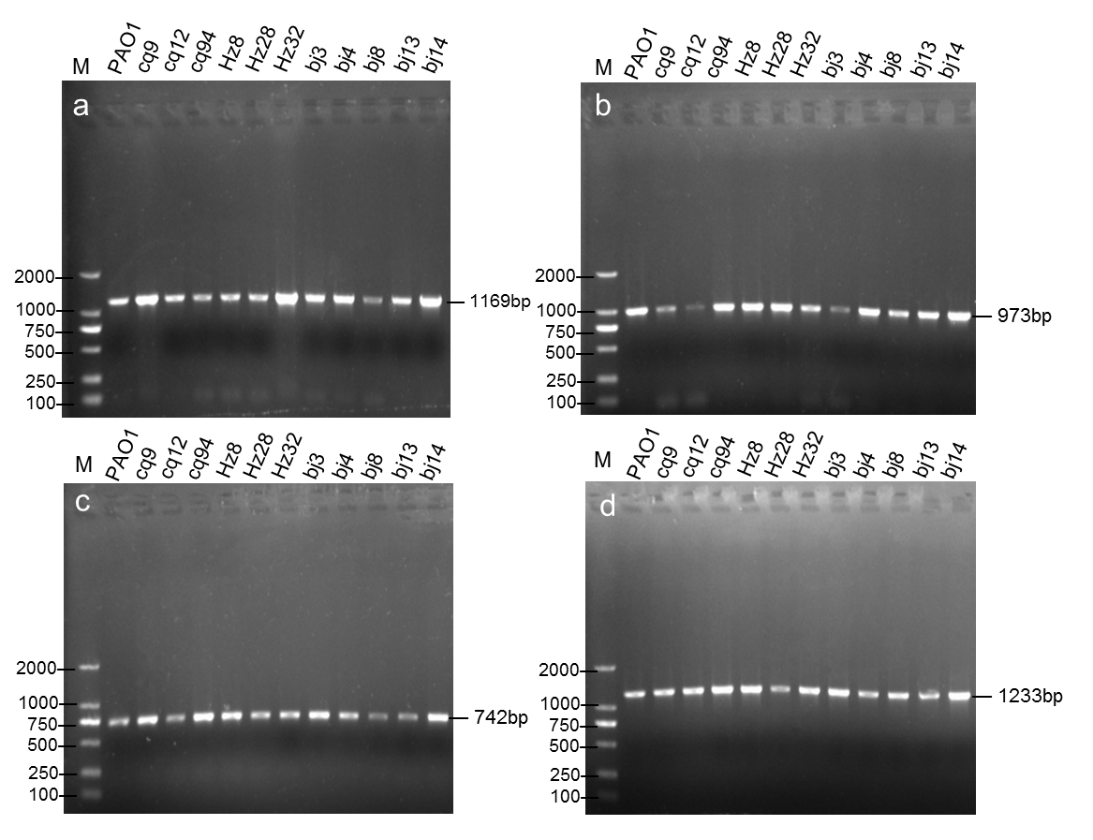
**

**Figure S1** Analysis of AHL-related QS genes in *P. aeruginosa* using PCR. (a) *lasI*, (b) *lasR*, (c) *rhlI*, (d) *rhlR*. Showing are agarose gel electrophoresis of PCR products amplified from the genomic DNA of biofilm-deficient *P. aeruginosa* isolates. PAO1 was used as the control. Primer parirs used are listed in Table S2. M: DNA marker. The size of the PCR products was shown.

**
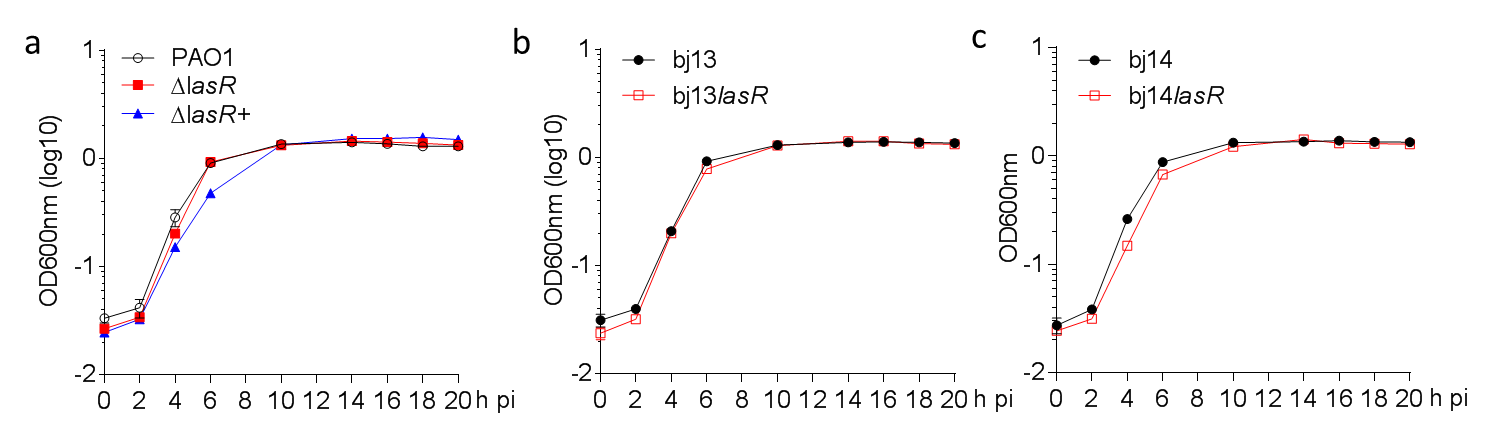
**

**Figure S2**. Comparison of *P. aeruginosa* growth curve. (**a**) PAO1, *ΔlasR,* and *ΔlasR+.* (**b**) bj13 and bj13*lasR.* (**c**) bj13 and bj13*lasR.* Overnight cultures of *P. aeruginosa* were inoculated into the fresh LB broth with a ratio 1:100 and cultured at 37 C. Bacteria were incubated at 37 °C with a shaking speed 250 rpm.

Bacterial growth were monitored by measuring the absorbance by spectrophotometry at 600 nm with sampling at two hours intervals.


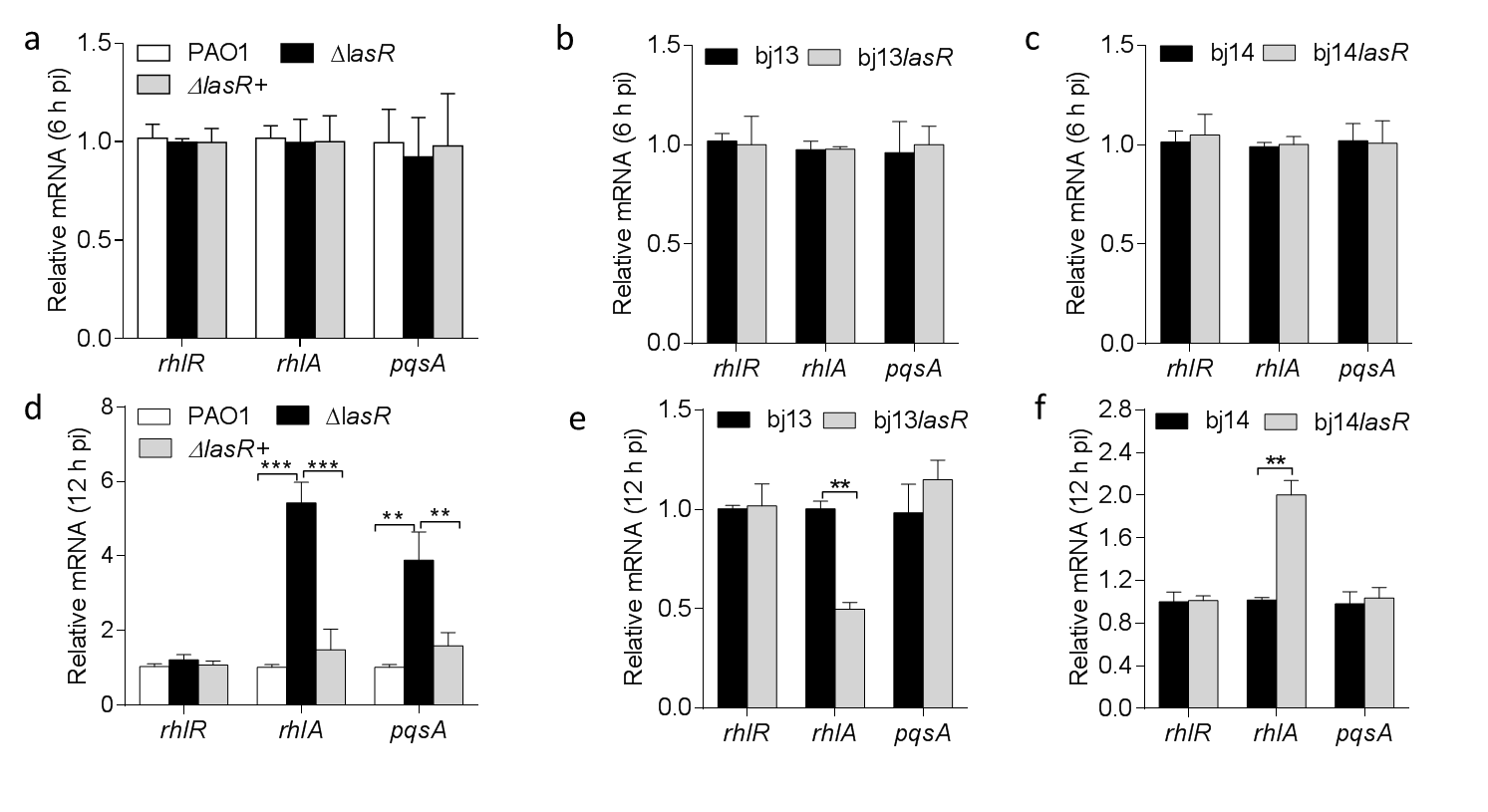


**Figure S3**. The growth-phase differential expression of *rhlR*, *rhlA* and *pqsA* genes in *P. aeruginosa.* (**a-c**) relative mRNA levels in bacteria grown for 6 hours. (**d-f**) relative mRNA levels in bacteria grown for 12 hours. The total RNAs from *P. aeruginosa* organisms were used for real time qRT-qPCR analysis. Relative mRNA amounts of *rhlR*, *rhlA,* and *pqsA* were normalized to that of the *rplS*. The representative data from *P. aeruginosa* strains were reported as mean ± SD of quadruplicates in an experiment. Experiments were repeated for three times. ****P*<0.001; ***P*< 0.005. *P* values were obtained by one way ANOVA with Bonferroni test.


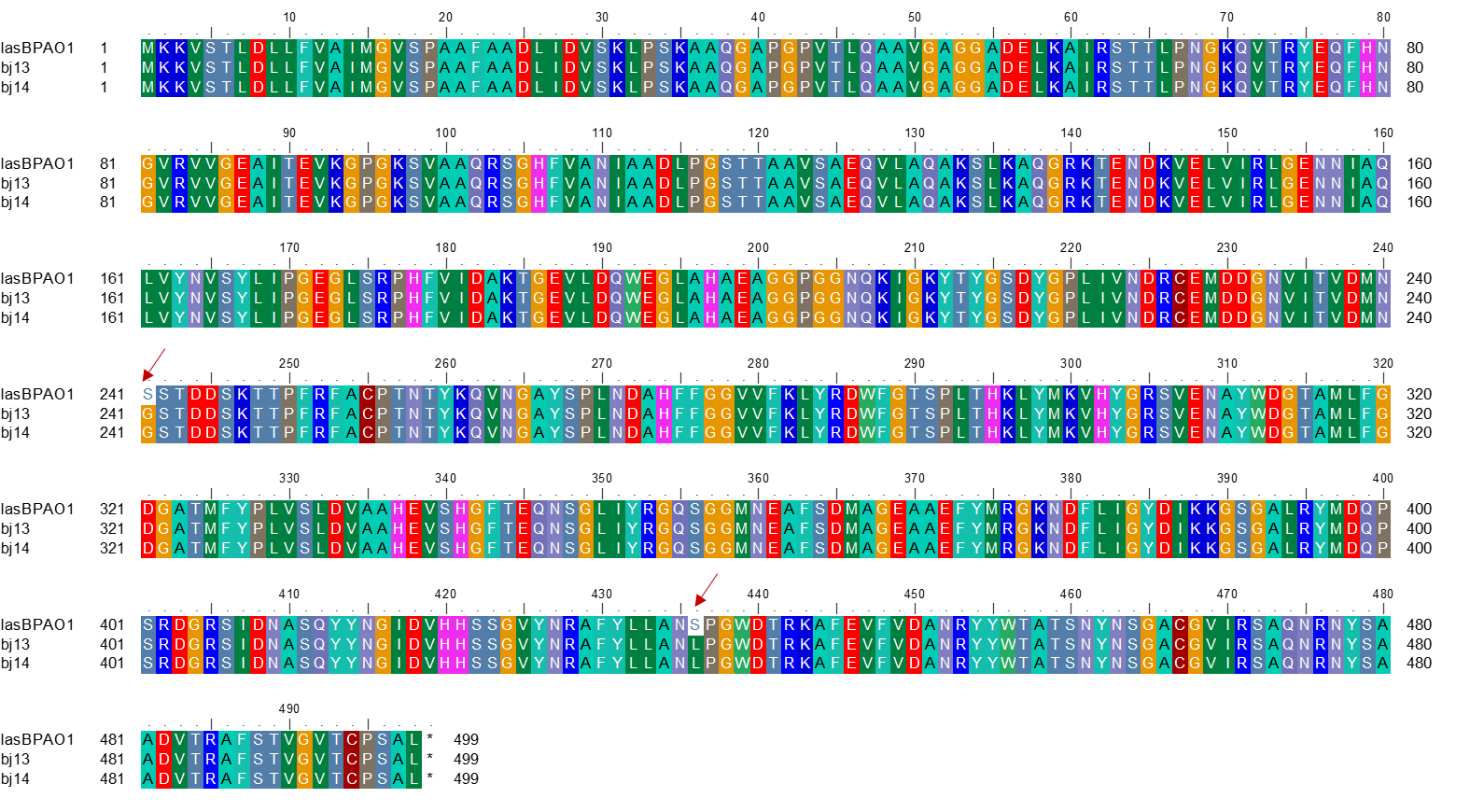


**Figure S4**. The alignment of LasB amino acid sequences in *P. aeruginosa isolates,* bj13 and bj14, using ClustalW. Arrows indicate the locations of amino acid changes in LasB.

**
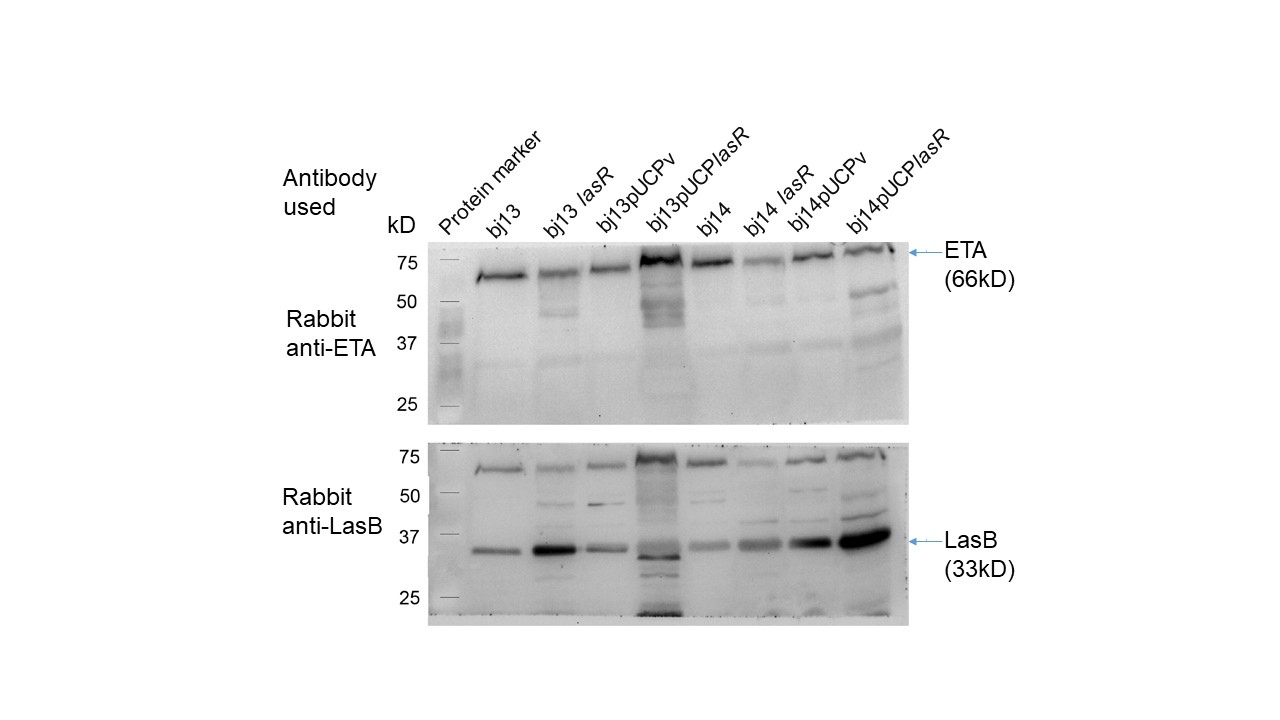
**

**Figure S5.** The full-length blot of LasB and ETA from the supernatant of *P. aeruginosa* cultures. The protein of interest was disclosed by an anti-ETA (upper panel) or anti-LasB (lower panel). Showings are the results from the isolates (bj13 and bj14), as well as the chromosomally *lasR*-complemented strains (bj13*lasR* and bj14*lasR*) (the same data are shown in Figure 5). In addition, the results from the strains harboring *lasR-*containing-plasmid, pUCP*lasR*, or empty vector, pUCPv, were also shown. To generate pUCP*lasR*, the *Kpn I-Hind III* cut PCR fragment containing the coding region and upstream 500 bps the translation start codon of *lasR* was ligated with the large fragment of *Kpn I-Hind III* digested pUCP-RedCm (Table S2)*.* Thus, *lasR* gene is under the control of its native promoters in pUCP*lasR*. pUCPv was derived from pUCP*lasR* by removing the Eco*RV*-digested fragment containing *lasR* coding region. The pUCPv or pUCP*lasR* was electroporated into bj13 or bj14, resulting in strains, bj13pUCP*lasR*, bj13pUCPv, bj14pUCP*lasR*, and bj14pUCPv, respectively.

**References**

1 Cowell, B. A., Twining, S. S., Hobden, J. A., Kwong, M. S. F. & Fleiszig, S. M. J. Mutation of lasA and lasB reduces Pseudomonas aeruginosa invasion of epithelial cells. *Microbiology (Reading, England)* **149**, 2291-2299, doi:doi:10.1099/mic.0.26280-0 (2003).

2 Schuster, M., Lostroh, C. P., Ogi, T. & Greenberg, E. P. Identification, timing, and signal specificity of Pseudomonas aeruginosa quorum-controlled genes: a transcriptome analysis. *Journal of bacteriology* **185**, 2066-2079 (2003).

3 Winson, M. K. *et al.* Construction and analysis of luxCDABE-based plasmid sensors for investigating N-acyl homoserine lactone-mediated quorum sensing. *FEMS microbiology letters* **163**, 185-192 (1998).

4 Yu, H. *et al.* Ndk, a novel host-responsive regulator, negatively regulates bacterial virulence through quorum sensing in Pseudomonas aeruginosa. *Scientific reports* **6**, 28684, doi:10.1038/srep28684 (2016).

5 Rao, X. *et al.* A regulator from *Chlamydia trachomatis* modulates the activity of RNA polymerase through direct interaction with the beta subunit and the primary sigma subunit. *Genes & development* **23**, 1818-1829, doi:10.1101/gad.1784009 (2009).

6 Bolivar, F. *et al.* Construction and characterization of new cloning vehicles. II. A multipurpose cloning system. 1977. *Biotechnology (Reading, Mass.)* **24**, 153-171 (1992).
